# Differential humoral and cellular immunity induced by vaccination using plasmid DNA and protein recombinant expressing the NS3 protein of dengue virus type 3

**DOI:** 10.1186/s12929-016-0302-z

**Published:** 2016-12-01

**Authors:** M. L. Hurtado-Melgoza, A. Ramos-Ligonio, L. M. Álvarez-Rodríguez, T. Meza-Menchaca, A. López-Monteon

**Affiliations:** 1 Doctorado en Ciencias Biomédicas, Universidad Veracruzana, Xalapa, Veracruz Mexico; 2 LADISER Inmunología y Biología Molecular, Facultad de Ciencias Químicas, Universidad Veracruzana, Orizaba, Veracruz Mexico; 3 Centro de Investigaciones Biomédicas, Universidad Veracruzana, Xalapa, Veracruz Mexico; 4 Facultad de Medicina, Universidad Veracruzana, Xalapa, Veracruz Mexico

**Keywords:** Dengue virus, NS3 protein, DNA vaccine, Recombinant protein

## Abstract

**Background:**

The dengue non-structural 3 (NS3) is a multifunctional protein, containing a serine-protease domain, located at the N-terminal portion, and helicase, NTPase and RTPase domains present in the C-terminal region. This protein is considered the main target for CD4+ and CD8+ T cell responses during dengue infection, which may be involved in protection. However, few studies have been undertaken evaluating the use of this protein as a protective antigen against dengue, as well as other flavivirus. In the present work we evaluated the potential of the NS3 (protease domain) as a protective antigen by comparing the administration of a recombinant protein versus a DNA vaccine in the mouse model.

**Results:**

BALB/c mice were immunized with the recombinant protein NS3-DEN3 via intraperitoneal and with plasmid pcDNA3/NS3-DEN3 intramuscularly and the immune response was evaluated. The activity of T lymphocytes was analyzed by the MTT assay, and cells of mice immunized with the recombinant protein showed no activity when stimulated with the homologous protein. However, cells from mice immunized with DNA, responded to stimulation with the recombinant protein. When the expression (RT-PCR) and cytokine production (ELISA) was evaluated in the splenocytes, different behavior depending on the type of immunization was observed, splenocytes of mice immunized with the recombinant protein expressed cytokines such as IL-4, IL-10 and produced high concentrations of IL-1, IL-6 and TNFα. Splenocytes from mice immunized with DNA expressed IL-2 and IFNγ and did not produce IL-6. In addition, immunization with the recombinant protein induced the production of antibodies that are detected up to a dilution 1:3200 by ELISA and Western blot assays, however, the serum of mice immunized with DNA presented no detectable antibody titers.

**Conclusion:**

The results obtained in this study show that administration of pcDNA3/NS3-DEN3 induces a favorable response in the activation of T lymphocytes with low production of specific antibodies against NS3-DEN3.

## Background

Dengue is the most prevalent arboviral disease worldwide. The outcome of the infection is determined by the interplay of viral and host factors. There are estimated 390 million dengue infections per year worldwide, of which 96 million manifest clinically with some level of severity [1]. The etiological agent of dengue disease is the Dengue virus (DENV) being the principal arthropod-borne viral pathogen affecting human populations. It is an enveloped virus member of the Flaviviridae family, containing a ~11 kb genome of positive single-stranded RNA which encodes three structural proteins (C, pr-M, E) and seven nonstructural proteins (NS1, NS2A, NS2B, NS3, NS4A, NS4B, NS5) [2]. Four serotypes of dengue virus (DENV-1, DENV-2, DENV-3, and DENV-4) cause dengue fever (DF) and more severe manifestations such as dengue hemorrhagic fever (DHF) and dengue shock syndrome (DSS) [3]. Currently, the infection caused by dengue virus is classified into dengue with/without warning signs and severe dengue [4].

Dengue virus NS3 is a multifunctional protein playing a major role during viral replication. Both protease and helicase domains of NS3 are interacting with human and insect host proteins including innate immune components of the host machinery [5]. The NS3 is a conserved protein among the different dengue serotypes, which elicits a strong cellular immune response after viral infection in humans and animal models [6, 7]. Nevertheless, there are only few studies evaluating the use of the NS3 protein as a protective antigen against DENV [8], as well as other virus from the Flaviviridae family [9]. In general, immunization with NS3 induced little protection in different animal models [10]. Our group has previously reported the expression of a fusion protein derived from the N-terminal region of the dengue virus NS3 protein for diagnostic use [11], however, the majority of T-cell epitopes are concentrated within the NS3 protein, the main target for CD4+ and CD8+ T cell response [6, 7, 11]. The role of T cells in immunity against dengue infection has been extensively reviewed [12, 13], the CD4+ T cell response contributes to protection by instructing B cell responses against the virus [14]. DNA vaccines are able to promote long-lasting cellular immunity against some pathogens, including flaviviruses [15]. Additionally, in contrast vaccination with recombinant protein, DNA vaccination approach promotes in vivo expression of antigens, as it originally occurs during the course of natural viral infections. Finally, DNA vaccines lead to suitable post-translational modifications and proper protein folding, features that can directly influence the quality of the elicited immunity [16–19]. Antigen expression in host cells can induce complete and lasting immune response including antibodies, although it is often weaker than that available with recombinant vaccines, as well as a strong and lasting activation of T helper cells and cytotoxic or cellular response [17, 20]. In this paper it was compared the immune response from a recombinant protein and a DNA vaccine derived from the NS3 protein (protease domain) in the mouse model.

## Methods

### Dengue virus and mice

Viral RNA of DEN3 (strain H87) was obtained by extracting 2 mL of the clarified culture media with 1 mL of TRIzol LS Reagent (Invitrogen) according to the manufacturer’s instructions and used as template for the synthesis of a cDNA and obtain the PCR product (590 pb) for the protease domain of NS3 protein according to that reported previously [11]. Mice of the BALB/c strain were purchased from CINVESTAV-IPN (Mexico, DF). All mice were maintained according to the recommendations of our Institutional Animal Care and Use Committee.

### Expression, Solubilization, and Purification of GST-NS3DEN3

Competent *Escherichia coli* strain DH5-α cells were transformed with the parental vector (pGEX-5X-1) and with the recombinant expression vector (pGEX-NS3DEN3), and were inoculated into LB media containing 100 mg/L ampicillin (Sigma, St. Louis, MO, USA), and incubated at 37 °C overnight. Fresh LB media was incubated at 37 °C with the overnight culture (1:100) to an OD_600_ = of 0.5, and protein production was induced by addition of isopropyl-β-D-thiogalactoside (IPTG) to a final concentration of 0.1 mM. After 2-h incubation, cells were harvested and purification of expressed proteins was performed essentially as described by López-Monteon et al. 2003 [17]. with the following modifications: Pellets were treated to solubilize the inclusion bodies; briefly, the pellets were washed twice with 50 mL of PBS (137 mM NaCl, 2.7 mM KCl, 4.3 mM Na_2_HPO_4_ · 7H_2_O, and 1.4 mM KH_2_PO_4_, pH 7.4), incubated at 37 °C under constant stirring for 20 min and centrifuged at 3046 × *g* at 4 °C for 10 min. After that, pellets were suspended by vortexing with PBS 1X pH 7.4 containing 2 M urea, the sample was stirred vigorously for 2 min, incubated at 37 °C under constant stirring for 30 min, and subsequently, centrifuged at 3046 × *g* for 10 min. The supernatants obtained from the solubilization of the inclusion bodies were dialyzed to remove urea. These supernatants were dialyzed against PBS 1X pH 7.4 overnight at 4 °C with constant stirring. The supernatant containing solubilized fusion protein (GST-NS3-DEN3) was mixed with glutathione-agarose beads (sulfur linkage; Sigma). After absorption for 30 min, beads were collected and washed by centrifugation. Either GST or GST-NS3-DEN3 were eluted by competition with free glutathione (15 mM glutathione in 50 mM Tris-HCl pH 8.0) and then acetone-precipitated.

### Purification of plasmid DNA

DNA plasmids pcDNA3 and pcDNA3/NS3-DEN3 were isolated from bacteria by alkaline lysis. Briefly, bacterial pellets were resuspended in 100 μL (25 mM Tris-HCl (pH 8.0), 10 mM EDTA (pH 8.0) and 50 mM saccharose) and repelleted. Alkaline lysis was performed by overlaying the pellets with 200 μL (0.2 N NaOH, 1% SDS), neutralization was achieved by adding 150 μL potassium acetate (5 M). The supernatant was extracted with phenol/chloroform, followed by ethanol precipitation of plasmid DNA. The purified DNA was run on 1% agarose gel in TAE buffer (45 mM Tris-acetic acid, 0.5 M EDTA, pH 8.0), and DNA bands were visualized by ethidium bromide staining.

### Cell treatment and transfection

HeLa cells were obtained from the American Type Culture Collection and were cultured in 1640 medium (Gibco, Life technology, USA) supplemented with 10% fetal bovine serum (Hyclone, Thermo Scientific, USA) in a humidified incubator in 5% CO_2_ at 37 °C. For transfection of pcDNA and pcDNA3/NS3DEN3, 1 × 10^5^ HeLa cells were seeded on each well of 24-well plate. After culturing for 24 h or adherent HeLa cells reached approximately 70% confluency, cells were transiently transfected under optimized transfection conditions. Briefly, 0.8 μg of plasmid DNA was diluted in 50 μL of OptiPro™SFM, and mixed with 2.0 μL of Lipofectamine® 2000 CD (Lipofectamine, Invitrogen, USA) in 50 μL of OptiPro™SFM and incubated for 20 min at room temperature. The mixture was then added to the cells, and incubated at 37 °C in a humidified atmosphere and 5% CO_2_ for further 72 h.

### Immunization of mice with recombinant protein and plasmid DNA

**Fig. 1:**
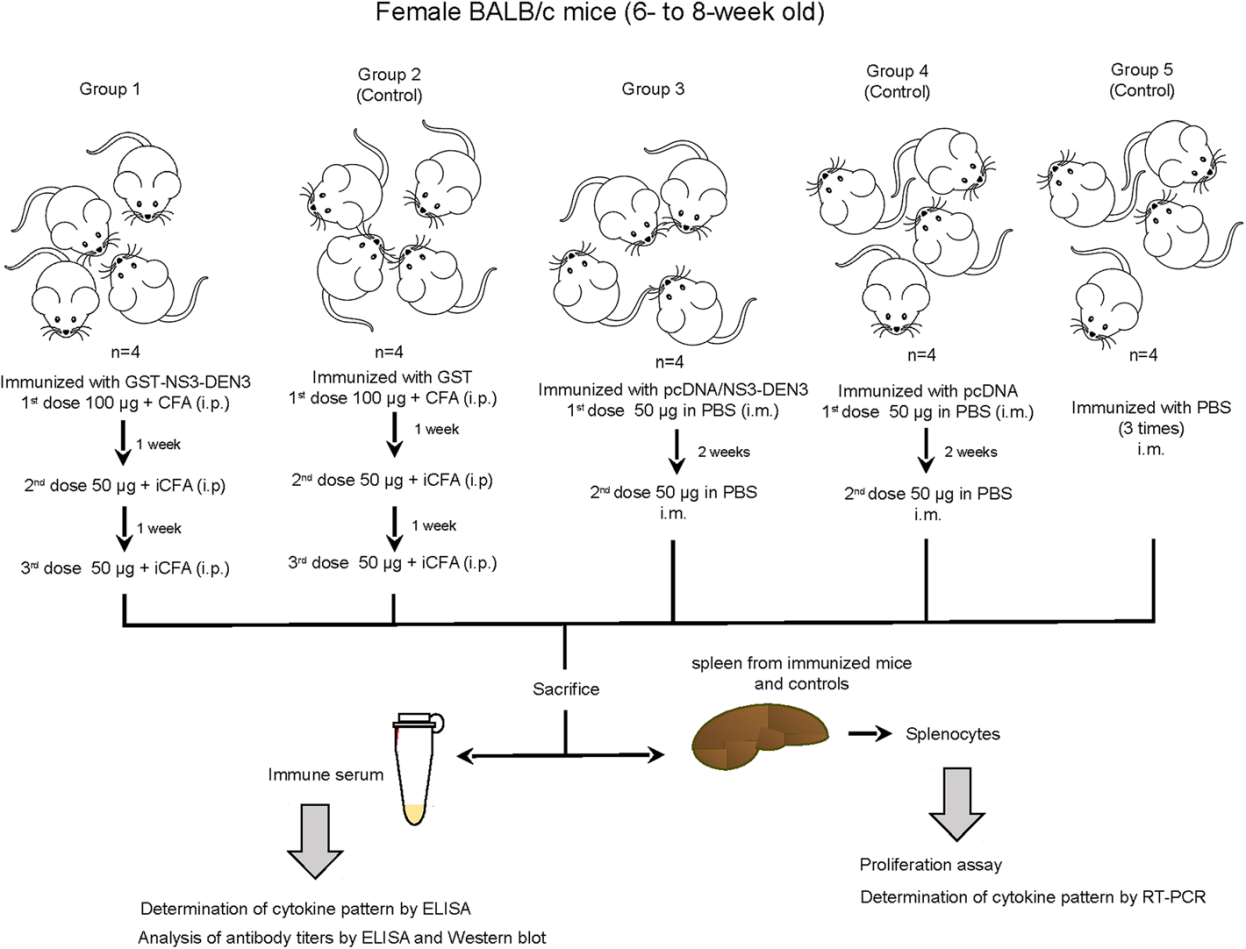
Schematic representation of the immunization schedule. Groups of BALB/c mice (*n* = 4) received three doses of GST-NS3-DEN3 or GST by the intraperitoneal (i.p.) route with one week of interval between each dose, or two doses of pcDNA/NS3-DEN3 or pcDNA by the intramuscular (i.m.) route with two weeks of interval between each dose. Two weeks after the third and second dose respectively, spleen and antiserum samples were collected from these animals. Splenocytes obtained were used for proliferation assays and determination of cytokines by RT-PCR. Moreover, the immune serum is used for determination of cytokines by ELISA and to analyze the antibody titer anti-NS3. To control the experiment, the same procedure was performed in parallel with splenocytes collected from non-vaccinated mice (*n* = 4)

Female BALB/c mice (6- to 8-week old) were immunized by the intraperitoneal route. The mice were immunized with one dose of 100 μg of GST-NS3-DEN3 and two more with 50 μg. First immunizations were performed with the antigen emulsified in complete Freund’s adjuvant (CFA), and re-immunizations at one-week intervals were performed with incomplete Freund’s adjuvant (Gibco-BRL, Grand Island, NY, USA). The same schedule was used for control group (*n* = 4), which received only GST plus adjuvant. At the end of the immunization scheme, animals were bled to obtain immune sera. On the other hand, DNA immunizations were done twice at 2-week intervals in each *tibialis anterioris* muscles with 50 μg of plasmids (pcDNA/NS3-DEN3 or parental pcDNA3) dissolved in 50 μL of phosphate buffer saline (PBS) (100 μg/mouse) (Fig. 1).

### Proliferation assay

Spleens from normal and immunized BALB/c mice were removed aseptically, dispersed into single-cell suspensions, and erythrocytes removed by treating cell suspensions with ACK buffer (150 mM NH_4_Cl, 1 mM KHCO_3_, 0.1 mM Na_2_EDTA pH 7.4) [21]. Splenocytes were resuspended in Dulbecco modified Eagle Medium (DMEM) containing 10% heat inactivated FCS (Gibco, BRL), 100 U/mL penicillin, 100 μg/mL streptomycin, 2 mM L-glutamine, and 0.05 mM 2-β-Mercaptoethanol. Spleen cells were cultured in 96-well flat-bottom plates at 4 × 10^5^/well and 37 °C under humidified atmosphere of 5% CO_2_. Cultures were stimulated in triplicate with 4 μg/mL of Concanavalin A (Con A, Sigma Chemical Co.), and with the antigen (an optimal dose of 10 μg/mL and 50 μg/mL was used throughout the experiments) in a total volume of 200 μL culture medium, at the end of culture, cells were centrifuged at 120 × *g* and resuspended in 100 μL of fresh medium, subsequently it was added 10 μL of the 12 mM MTT [3-(4,5-dimethylthiazol-2-yl)-2,5-diphenyltetrazolium bromide] stock solution (Vybrant®, Molecular Probes) to each well. Include a negative control of 10 μl of the MTT stock solution added to 100 μL of medium alone. The plate was incubated at 37 °C for 4 h. At the end of the incubation, 100 μL of the SDS-HCl solution were added to each well and mixed thoroughly using the pipette and the plate was incubated at 37 °C for 4–18 h in a humidified chamber. Finally, samples were mixed using a pipette and absorbance read at 570 nm in a microplate reader (Multiskan EX, Thermo Electron Corporation).

### RNA Isolation and RT-PCR

Total RNA from cells, cultured in 24-well plates with different treatments for 48 h, was isolated using the TRIzol system (Life Technologies). One microgram of RNA was reverse transcribed to cDNA with an oligonucleotide (poly(dT)_16_) using the SuperScript II reverse transcriptase (Life Technologies) and the cDNA used as a template for PCR. PCR sequences and PCR conditions used for amplification of GAPDH, IL-2 [22], IL-4, IL-10 and IFN-γ [23]: GAPDH (5′-GGT GAA GGT CGG AGT CAA CGG-3′ and 5′-GGT CAT GAG TCC TTC CAC GAT-3′), IL-2 (5′-GAC ACT TGT GCT CCT TGT CA-3′ and 5′- TCA ATT CTG TGG CCT GCT TG -3′), IL-4 (5′- ATG GGT CTC AAC CCC CAG CTA GT -3′ and 5′- GCT CTT TAG GCT TTC CAG GAA GTC - 3′), IL-10 (5′- CCT GGT AGA AGT GAT GCC CCA GGC A −3′ and 5′- CTA TGC AGT TGA TGA AGA TGT CAA A −3′), IFN-γ (5′- TGA ACG CTA CAC ACT GCA TCT TGG −3′ and 5′- TGA CTC CTT TTC CGC TTC CTG AG −3′). PCR conditions were as follows: initial DNA denaturation at 94 °C for 5 min and 35 rounds of denaturation: 95 °C for 1 min, annealing (55 °C for IL-2; 60 °C for IL-4, IL-10, and IFNγ, and 59 °C for GAPDH), and extension 72 °C for 1 min. PCR products were electrophoresed on 1.8% agarose gels containing 0.5 μg/mL ethidium bromide and photographed under ultraviolet light.

### Determination of cytokine pattern by ELISA

The interleukin 1-beta (IL-1β), IL-6 and TNF-α were quantified by ELISA in culture supernatants of splenocytes under different conditions of stimulation (120 h), according to the manufacturer’s protocol. Briefly, 96-well flat-bottom plates were coated overnight with a capture antibody at a final concentration of 2 μg/mL, and then plates were blocked with 10% PBS-FCS, washed three times, and incubated with the cell culture supernatant samples or control antigens overnight at 4 °C. After washing, plates were incubated with the respective biotinilated anti-cytokine antibodies (R&D System) at 1 μg/mL for 1 h in the dark. Plates were washed and Streptavidin-Alkaline Phosphatase at 1:2000 was added for 30 min in the dark, then washed, and 100 μL of ABTS (2,2,-azino-bis (3-ethylbenzthiazoline) 6-sulphonic acid) (Life Technologies) was added as substrate and the reaction was allowed to proceed for 20 min at room temperature (RT); the reaction was stopped with 2% sulphuric acid, and absorbance was read at 415 nm by an ELISA reader (MultiskanMS, Labsystem).

### SDS-PAGE and Western blot analysis

Recombinant protein NS3-DEN3 and/or the total extract of cells transfected with plasmid pCDNA3/NS3-DEN3 were resolved on 10% SDS-PAGE [24] and visualized by staining with Coomassie brilliant blue or electrophoretically transferred onto nitrocellulose paper for immunoblotting [25, 26]. Blots were incubated with an anti-NS3 antibody at 1:2000 dilution, detected using alkaline phosphatase-goat anti-mouse IgG (Pierce, Rockford, IL, USA) diluted at 1:5000, then developed with NBT and BCIP (Sigma).

### Analysis of antibody titers by ELISA and Western blot

ELISA plates were coated overnight at 4 °C with 2 μg/mL of NS3-DEN3 recombinant protein, in carbonate buffer (pH 9.6). The plates were washed six times with PBS containing 0.1% Tween (PBST) and incubated for 2 h at 37 °C with blocking solution (PBS containing 5% skim milk). Plates were then washed three times with PBST, three times with PBS, and incubated with 50 μL of mouse anti-GST-NS3-DEN3 as primary antibodies at serial dilutions (1:100 – 1:3200) in PBST, and incubated for 2 h at room temperature. Further washing steps were conducted and a peroxidase- labeled goat anti-human IgG antibody (Pierce, Rockford, IL) was added at 1:8000 dilution in PBS/0.05% Tween 20 and incubated for 1 h at room temperature. After six washes, 100 μL of 2,2,-azino-bis (3-ethylbenzthiazoline)-6-sulphonic acid (Zymed, South San Francisco, CA) was added as substrate and the reaction was allowed to proceed for 20 min at room temperature. The reaction was stopped with 2% sulfuric acid, and absorbance was read at 405 nm with an ELISA microplate reader (Multiscan MS; Labsystems, Vantaa, Finland).

Pooled sera from each group of immunized mice were used as primary antibodies at serial dilutions (1:100–1:3200) in TBS-T (150 mM NaCl, 0.05% Tween 20, 2% skim milk, and 10 mM Tris–HCl pH 7.4). Bound antibodies were detected using alkaline phosphatase-conjugated goat anti-mouse IgG (Pierce, Rockford, IL, USA) diluted at 1:5000, then developed with NBT and BCIP (Sigma).

### Statistical analysis

Statistical analysis was performed with GraphPad Prism (Version 5.0). The results are presented as mean ± standard deviation. Analysis of variance (ANOVA) followed by Tukey’s post-hoc test was performed to compare the mean values among various groups. A *p* value of <0.05 was considered statistically significant.

## Results

### Plasmid construction, expression and transfection

**Fig. 2:**
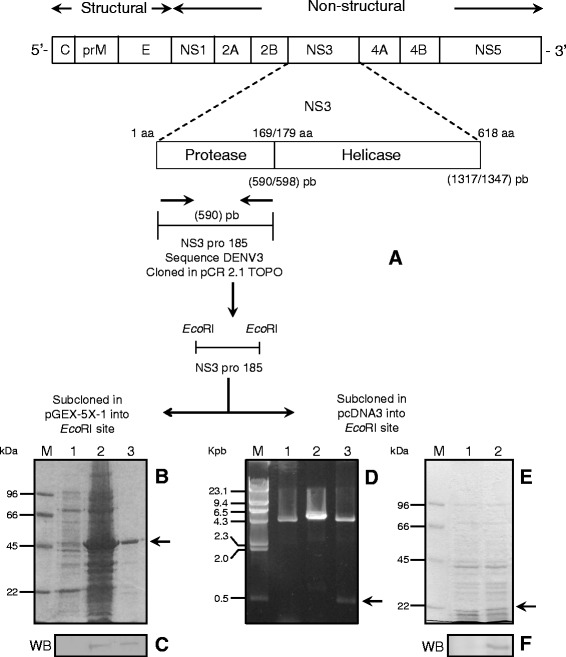
Obtaining recombinants plasmids and recombinant NS3 protein. **a** Cloning strategy for the NS3 domain protease of dengue virus in the cloning vector and in prokaryotic and eukaryotic expression vector. **b** Purification of the recombinant protein NS3DEN3. SDS-PAGE 10% coomassie blue stained. (M) molecular weight marker. (1) Total cell extract of *E. coli* DH5α uninduced. (2) Total cell extract of *E. coli* DH5α induced with IPTG. (3) recombinant NS3DEN3 protein. The *arrow* points to the purified protein NS3DEN3 (**c**) Detection of the recombinant NS3 protein by western blot with an anti-NS3 antibody. **d** (M) size marker. (Lane 1) pcDNA3 plasmid linearized with *Eco*RI. (Lane 2) recombinant plasmid pcDNA3/NS3DEN3 linearized with *Eco*RI (Lane 3) Fragment release NS3 pro-185. The arrow indicates the fragment released. **e** HeLa cells transfected. SDS-PAGE 10% coomassie blue stained. (M) molecular weight marker. (1) Total extract untransfected HeLa cells. (2) total extract of HeLa cells transfected with plasmid pcDNA3/NS3/DEN3. **f** Detection of NS3 protein by western blot with an anti-NS3 antibody

A plasmid based on the pCR 2.1-TOPO vector (Invitrogen-Life Technologies) was constructed encoding the NS3pro185 sequence (domain protease of NS3). The recombinant vector was restricted with *Eco* RI, and the fragment was ligated in frame into pGEX-5X-1 (Pharmacia) [11] and pcDNA3 (Invitrogene-Life Technologies) vectors previously digested with this same enzyme. Competent *Escherichia coli* strain DH5-α cells were transformed with the parental vector (pGEX-5X-1) as with the recombinant expression vector (pGEX-NS3-DEN3) (Fig. 2a). The protein production was induced by addition of isopropyl-β-D-thiogalactoside (IPTG). After incubation, cells were harvested and purification of expressed protein (GST-NS3-DEN3) was performed as previously described [17] (Fig. 2b). DNA plasmids pcDNA3 and pcDNA3/NS3-DEN3 were isolated from bacteria by alkaline lysis procedure, and DNA bands were visualized by ethidium bromide staining (Fig. 2d). HeLa cells were transiently transfected with recombinant plasmid (pcDNA3/NS3-DEN3) or control vector (pcDNA3) using lipofectamine, and harvested 18 h after transfection. Cell extracts of the transfected cells and untransfected were resolved on 10% SDS-PAGE (Fig. 2e), and transferred into nitrocellulose membranes. Recombinant protein was detected with polyclonal antibodies against the GST-NS3-DEN3 protein (Fig. 2c and f).

### pcDNA3/NS3-DEN3 induces proliferation but not the protein NS3-DEN3

**Fig. 3:**
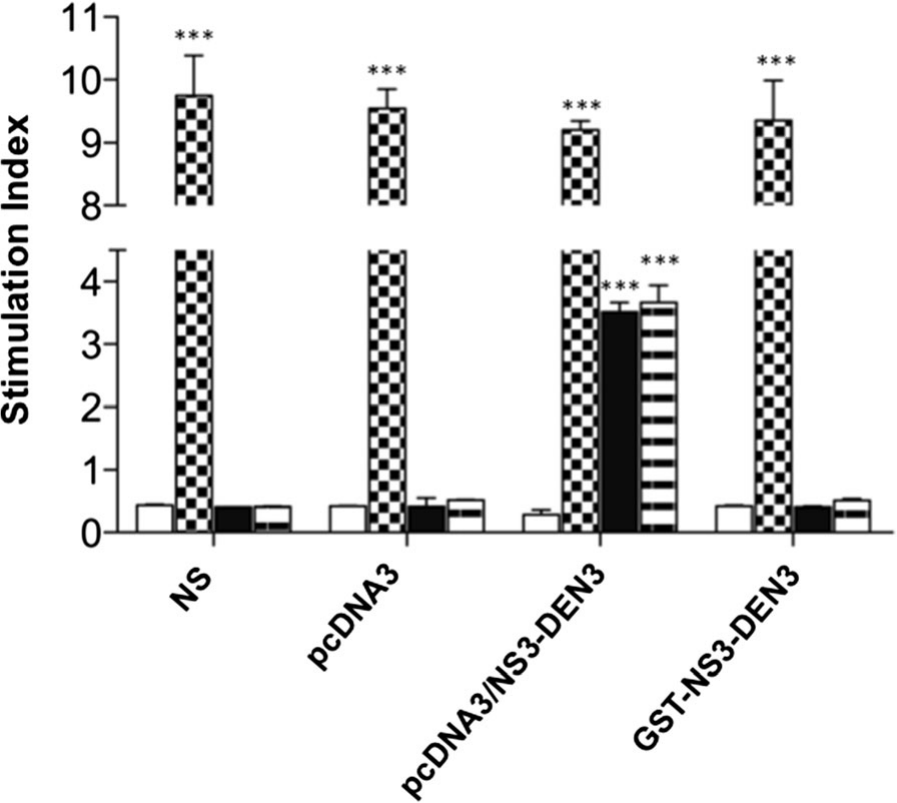
Splenic cell proliferation upon stimulation with NS3-DEN3. The MTT assay was performed with splenocytes from normal mice or pcDNA3, pcDNA3/NS3DEN3, and NS3DEN3 immunized mice (cells collected 7 days after the last immunization, three times at one-week intervals). Non-stimulated (NS) (*White bars*) or stimulated with Con A (*checkered bars*), NS3DEN3 10 μg/mL (*Black bars*), or NS3DEN3 50 μg/mL (*striped bars*). (***P < 0 · 0001)

The activation of T cell was evaluated by administration of the antigen in the mouse model. To analyze the ability to induce proliferation of NS3-DEN3 protein, splenocytes from non-immunized and immunized animals with the recombinant protein NS3-DEN3 and plasmid pcDNA3/NS3-DEN were evaluated. The proliferative responses by spleen cells from normal and immunized mice (with plasmid and/or recombinant protein) stimulated in vitro with Con A (72 h), GST-NS3-DEN3 10 and 50 μg/mL (120 h) and GST 10 and 50 μg/mL (Data not shown). Con A induced proliferation of splenic T cells from normal as well as from immunized mice, no response was observed when splenocytes from animals normal or immunized with the recombinant protein were stimulated with GST-NS3DEN3, however, cells of mice immunized with the plasmid showed a significant response towards the stimulation with the recombinant protein (Fig. 3).

### NS3 induced differential expression of genes and production of cytokines depending on the method of administration

**Fig. 4:**
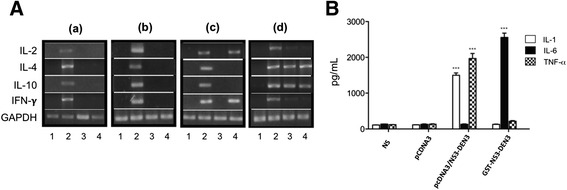
Effect of immunization with pcDNA3/NS3DEN3 and recombinant NS3DEN3 protein on cytokine gene expression in splenocytes of BALB/c mice. **a** RT-PCR analysis of cytokines mRNA in splenocytes (*a*) Normal mice, (*b*) mice immunized with pcDNA3, (*c*) mice immunized with pcDNA3/NS3DEN3, and *d*) mice immunized with NS3DEN3. (Lane 1) non-stimulated cells, or stimulated (Lane 2) Con A, (Lane 3) NS3DEN3 10 μg/mL, (Lane 4) NS3DEN3 50 μg/mL. GAPDH was used as control housekeeping gene. **b** Profile of cytokines induced by NS3DEN3 in splenocytes. Splenocytes from immunized mice and normal mice were stimulated with the NS3DEN3 protein for 120 h, and cytokines was measured in cells culture supernatants by ELISA. IL-1 (*open bars*), IL-6 (*full bars*), and TNFα (*checkered bars*). Histograms show values in pg/mL (means ± SD) of three experiments run in duplicate. *,**,***P < 0.05, 0.001, and 0.0001, respectively, versus unstimulated cells

It is known that pro-inflammatory and anti-inflammatory cytokines may contribute to the pathogenesis of the infection and affect the function of all cells types involved in an immune response. To investigate whether the method of administration of GST-NS3-DEN3 recombinant protein alters cytokine expression, RT-PCR analysis was performed in splenocytes from mice immunized with the recombinant protein and/or with the plasmid stimulated in vitro with GST-NS3-DEN3 recombinant protein (Fig. 4a). When splenocytes were stimulated with GST-NS3-DEN3 recombinant protein, an increase in the expression of genes for IL-4 and IFN-γ was observed in cells immunized with the plasmid and stimulated with the protein for 48 h (Fig. 4a(*c*), lane 4). However, cells from mice immunized with the recombinant protein do not express IL-2 or IFN-γ, but they express IL-4 and IL-10 (Fig. 4a(*d*), Lane 3 and 4). Furthermore, when the production of pro-inflammatory cytokines was analyzed in the in the culture supernatant of splenocytes there was an increase in the production of IL-1 and TNF-α in splenocytes from mice immunized with plasmid pcDNA3/NS3-DEN3 and not so in splenocytes from mice immunized with GST-NS3-DEN3 recombinant protein where only an increase in the production of IL-6 was observed (Fig. 4b).

### NS3-DEN3 protein induce the production of antibodies but not the DNA vaccine pcDNA3/NS3-DEN3

**Fig. 5:**
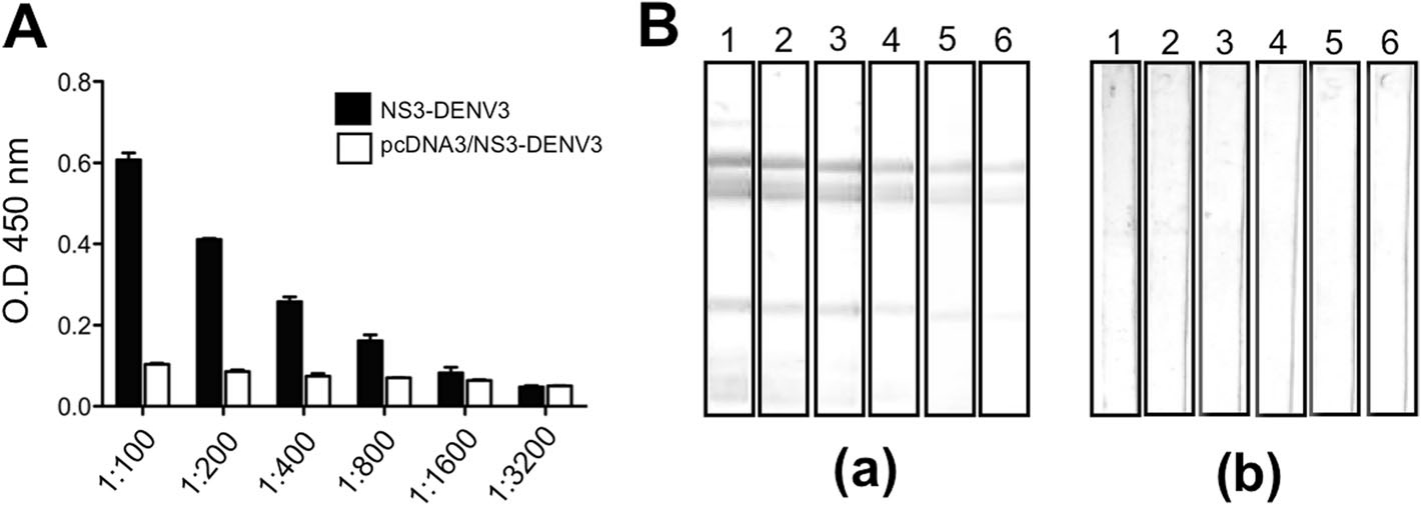
Analysis of antibodies against NS3. **a** Determination of anti-NS3 antibody titer. NS3DEN3 protein was applied to a 96-well ELISA plate, and anti-NS3 antibodies at different dilutions were added in different wells. Full bars denote the antibody titer obtained by immunization with the recombinant protein. Open bars denote the antibody titer obtained by immunization with plasmid pcDNA3/NS3DEN3. Pre-immune serum was used as a negative control. Bound antibody was determined using secondary antibody tagged with HRP. Each bar represents the mean ± standard error of the mean from three independent experiments. **b** Analysis of antibodies against NS3 by Western blotting. NS3DEN3 was run in SDS-PAGE, transferred to nitrocellulose membrane. The membrane was cut into strips, and each strip was developed with primary antibodies at different dilutions followed by alkaline phosphatase-conjugated secondary antibodies. (*a*) Serum obtained from mice immunized with the recombinant protein, and (*b*) serum obtained from mice immunized with DNA. Lane 1, 1:100; lane 2, 1:200; lane 3, 1:400; lane 4, 1:800; lane 5, 1:1600 and lane 6, 1:3200. A representative blot from three independent experiments is presented here

The humoral immune response against GST-NS3-DEN3 was evaluated by immunization of BALB/c mice. The sera collected from mice 7 days after the last immunization with recombinant protein or immunized with the plasmid pcDNA3/NS3-DEN3 were analyzed by ELISA and WB. The results showed that the antibodies obtained by immunization with the GST-NS3-DEN3 recombinant protein recognize their homologous protein at a dilution of 1:1600 by ELISA assays (Fig. 5a) and up to a dilution of 1:3200 by WB (Fig. 5b). However, the serum obtained from mice immunized with the plasmid pcDNA3/NS3-DEN3 was not able to recognize GST-NS3-DEN3 recombinant protein by the methods mentioned above (Fig. 5a, b).

## Discussion

The NS3 protein is a multifunctional non-structural protein of flaviviruses implicated in the polyprotein processing. The predominance of cytotoxic T cell lymphocytes epitopes on the NS3 protein suggests a protective role of this protein in limiting virus replication. The NS3 protein is vitally important for the virus since it is involved in activities such as virus replication, including protease, NTPase, helicase, and RNA binding and also capping of the viral genomic RNA [27]. Despite there are several reports on the structure and function of the NS3 protein, there are not many studies evaluating the use of the NS3 protein as an immunogenic antigen. In this work it was evaluated the immunogenicity and antigenicity of the recombinant protein GST-NS3-DEN3 and a recombinant plasmid pcDNA3/NS3-DEN3 derived from NS3 protein. It is known that most of dengue non-structural proteins expressed in bacterial cells have been found after cellular disruption in the insoluble fraction therefore denaturation and refolding processes are required to recover biologically active proteins [28, 29]. The protease region of NS3 protein was expressed in *E. coli* as inclusion bodies, which were subsequently purified. Moreover, a recombinant plasmid was obtained. The homogeneity of the purified GST-NS3-DEN3 recombinant protein as well as transfection efficiency were demonstrated by Western blot.

T cell activation is a critical event for an effective immune response against infection, including the production of cytokines [30]. How T cells contribute to the immune response in dengue disease, has not been clearly defined yet. The two-effector arms of the immune system relevant to protection from viral disease in humans are neutralizing antibodies and cytotoxic T lymphocytes. Because T cells do not recognize intact virions, dengue virus-specific T cells would not be able to provide sterilizing immunity against viral infection, nevertheless, DNA and recombinant virus-based vaccines were shown to induce protective immunity that involved both antibody and T cell responses [31, 32]. Our results show that splenocytes from mice immunized with DNA were able to respond to in vitro stimulation by the protein, and not cells from mice immunized with the recombinant protein. T lymphocytes are not infected by dengue virus, nevertheless it is possible that exposure to virus and/or proteins viral may affect their function. T lymphocytes from DENV-infected individuals are impaired in their proliferative capacity, although this effect has been attributed to altered function of antigen-presenting cells rather than to an intrinsic defect on T lymphocytes [33]. The host immune responses have been considered as the major factor responsible for dengue pathogenesis, resulting in an altered immune response, which triggers T cell activation, and the release of cytokines and chemical mediators has been a risk factor in secondary Infection. During infection occurs a ‘cytokine storm’, defined as an imbalance between cytokines driving an inflammation (pro-inflammatory) and those silencing an inflammation (anti-inflammatory). Therefore, serum cytokine and chemokine levels can serve as a laboratory tool for predicting severe disease [34]. As it was observed in the results, DNA immunization induces the synthesis of cytokines of Th1 profile which can activate T cells, however, the immunization with the recombinant protein induces a cytokine profile similar to that observed during virus infection, where a marked expression of IL-10 gene and production of IL-1, IL-6, and TNF-α is observed. Studies show that elevated levels of IL-6, IL-10, IFN-γ, MIF, and CCL-4 could be used as potential predictors of severe dengue [35, 36]. Also a model of a lethal DENV-3 infection, high levels of TNF-α and IL-6 were detected in the serum during the final stages of the disease [37]. Several lines of evidence indicate that displacement of viral genotype and host genetic background are key factors driving the production of a cytokine storm. Several cytokines are known to induce apoptosis, a form of cell suicide (cause of hemorrhage), and/or affect adherents junctions (cause permeability) in vitro. Whether these cytokines may have such effects in vivo remains to be established [38]. The development of a dengue virus (DENV) vaccine has been hampered by the requirement of simultaneous protection against four distinct serotypes and the threat that DENV-specific antibodies might either mediate neutralization or, on the contrary, exacerbate disease through the phenomenon of antibody-dependent enhancement (ADE) of infection. In this work, it was observed that immunization with plasmid did not induce detectable antibody titers, and not so immunization with the recombinant protein, this may be due to endogenous antigen expression within cells host where DNA immunization can induce an immune response complete and lasting, with presence of antibodies, although this presence is often weaker than that obtainable with recombinant proteins. It is thought that the efficacy of DNA vaccines is mainly due to the antigen mechanisms presentation involved [39]. DNA immunization has been used as a platform for developing a tetravalent dengue vaccine in response to the high priority as a public health problem, animal studies have succeeded in generating anti-dengue cellular and humoral immune responses that were protective either completely or partially against challenge with live dengue virus [40]. Finally, in other studies, it has been observed that the DNA vaccines encoding the full-length NS3 induced a cellular immune response against this epitope with the production of IFN-γ. Therefore, results suggest the participation of a cellular immune response in the mechanism of protection induced by the DNA vaccines based on the NS3 protein [41].

## Conclusions

If one considers that most vaccine candidates against DENV focus their attention on the induction of neutralizing antibodies against the virus, and that work on cellular immunity are limited, the results obtained in this study show that administration of pcDNA3/NS3-DEN3 induced a favorable response with the activation of T lymphocytes with low production of specific antibodies against NS3-DEN3. These results suggest the need to address the evaluation of the immune response, further studies will be necessary for the establishment of the mechanisms involved in the protection induced by the DNA vaccines based on the NS3 protein.

## Abbreviations

ABTS: (2,2,-azino-bis (3-ethylbenzthiazoline) 6-sulphonic acid
BCIP: 5-Bromo-4-chloro-3-indolyl phosphate
CFA: Complete Freund’s adjuvant
Con A: Concanavalin A
DENV: Dengue virus
DF: Dengue fever
DHF: Dengue hemorrhagic fever
DSS: Fever dengue shock syndrome
ELISA: Enzyme-Linked ImmunoSorbent Assay
FCS: Fetal calf serum
GAPDH: Glyceraldehyde-3-phosphate dehydrogenase
GST: Glutation -S-Transferase
GST-NS3-DEN3: Recombinant protein NS3 from serotype 3 coupled to GST
IFA: Incomplete Freund’s adjuvant
IPTG: Isopropyl-β-D-thiogalactoside
MTT: [3-(4,5-dimethylthiazol-2-yl)-2,5-diphenyltetrazolium bromide]
NBT: Nitroblue tetrazolium
PBS: Phosphate buffer solution
pcDNA3/NS3-DEN3: DNA vaccine
PCR: Polimerase chain reaction
RT-PCR: Retro transcription- PCR assay
SDS-PAGE: Sodium dodecyl sulfate polyacrylamide gel electrophoresis
